# Epigeic Earthworms Exert a Bottleneck Effect on Microbial Communities through Gut Associated Processes

**DOI:** 10.1371/journal.pone.0024786

**Published:** 2011-09-15

**Authors:** María Gómez-Brandón, Manuel Aira, Marta Lores, Jorge Domínguez

**Affiliations:** 1 Departamento de Ecoloxía e Bioloxía Animal, Facultade de Bioloxía, Universidade de Vigo, Vigo, Spain; 2 Laboratorio de Investigación y Desarrollo de Soluciones Analíticas. Departamento de Química Analítica, Facultad de Química, Santiago de Compostela, Spain; Argonne National Laboratory, United States of America

## Abstract

**Background:**

Earthworms play a critical role in organic matter decomposition because of the interactions they establish with microorganisms. The ingestion, digestion, assimilation of organic material in the gut and then casting is the first step in earthworm-microorganism interactions. The current knowledge of these direct effects is still limited for epigeic earthworm species, mainly those living in man-made environments. Here we tested whether and to what extent the earthworm *Eisenia andrei* is capable of altering the microbiological properties of fresh organic matter through gut associated processes; and if these direct effects are related to the earthworm diet.

**Methodology:**

To address these questions we determined the microbial community structure (phospholipid fatty acid profiles) and microbial activity (fluorescein diacetate hydrolysis) in the earthworm casts derived from three types of animal manure (cow, horse and pig manure), which differed in microbial composition.

**Principal Findings:**

The passage of the organic material through the gut of *E. andrei* reduced the total microbial biomass irrespective of the type of manure, and resulted in a decrease in bacterial biomass in all the manures; whilst leaving the fungi unaffected in the egested materials. However, unlike the microbial biomass, no such reduction was detected in the total microbial activity of cast samples derived from the pig manure. Moreover, no differences were found between cast samples derived from the different types of manure with regards to microbial community structure, which provides strong evidence for a bottleneck effect of worm digestion on microbial populations of the original material consumed.

**Conclusions/Significance:**

Our data reveal that earthworm gut is a major shaper of microbial communities, thereby favouring the existence of a reduced but more active microbial population in the egested materials, which is of great importance to understand how biotic interactions within the decomposer food web influence on nutrient cycling.

## Introduction

Epigeic earthworms are known to play a critical role in the decomposition of organic matter, thus significantly accelerating decomposition rates and nutrient turnover [1]. These earthworms live in the soil organic horizon and mainly feed on fresh organic matter contained in forest litter, litter mounds and herbivore dungs, as well as in man-made environments such as manure heaps [2]. Soil litter and manure heaps are hotspots of heterotrophic activity, in which earthworms interact closely with microorganisms and other organisms within the decomposer community [2]–[7], thus strongly affecting decomposition processes [8]. Epigeic earthworms directly affect the decomposition of soil through gut associated processes, i.e. via the effects of ingestion, digestion and assimilation of the organic matter and microorganisms, which are then released in earthworm casts [9], [10]. Specific microbial groups respond differently to the gut environment [11] and selective effects on the presence and abundance of microorganisms during the passage of organic material through the gut of these earthworm species have been observed. For instance, some bacteria are activated during passage through the gut, whereas others remain unaffected and others are digested in the intestinal tract and thus decrease in number [12]–[14]. Monroy *et al.* [14] observed a reduction in the density of total coliforms by 98%, after the passage of pig slurry through the gut of the epigeic earthworm *Eisenia fetida*. This reduction in total coliform numbers was not related to decreases in bacterial biomass C, which indicates a specific negative effect of the earthworms on this bacterial group. Accordingly, Pedersen & Hendriksen [12] reported a selective reduction of the coliform *Escherichia coli* BJ18 in cattle dung during the passage through the gut of several species of earthworms of the genus *Lumbricus*. This reduction did not affect other specific bacterial groups, resulting in shifts in the composition of the microbial community. The selective effects on ingested microbes through the earthworm gut may be caused by competitive interactions between those ingested and the endosymbiotic microbes that reside in the gut [15]; and/or by selective suppressive activity of gut fluids against specific microbial groups [16]. Thus, when comparing casts of *Lumbricus rubellus* to the surrounding soil, Furlong *et al.* [17] revealed that the abundance of Gammaproteobacteria, Firmicutes and Actinobacteria increased considerably as a result of gut passage from the bulk soil to the cast. Indeed, Knapp *et al.* [18] found a strong occurrence of Gammaproteobacteria in the gut microbiota of *L. rubellus*, irrespective of the type of food source ingested. Moreover, König *et al.* [19] observed that Proteobacteria, especially from the Gamma-subclass, are abundantly detected in the digestive tract of soil invertebrates followed by representatives of the phyla Firmicutes, Actinobacteria and the Bacteroides/Flavobacterium group. The distribution pattern of the earthworm gut microbiota at the host population level is of fundamental importance to understand host-microbiota interactions. Recently, Rudi *et al.* [20] observed a rapid and homogenous change of the microbiota in the gut of the epigeic earthworm *Eisenia hortensis* as a response to feeding. More specifically, they found a low-density and highly variable microbiota among the earthworms before feeding, while a high-density homologous microbiota resulted after one and seven days after feeding. Understanding these changes in gut associated microbiota will help to better understand the interplay between earthworms and microorganisms, and in turn the functional importance of earthworms on overall soil function through the gut associated processes. Changes in microbial communities due to the gut selective effects on the presence and abundance of ingested microbes may alter the decomposition pathways, probably by modifying the composition of the microbial communities involved in decomposition, as microbes from the gut are then released in faecal material where they continue to decompose egested organic matter. Indeed, epigeic earthworms are known to excrete large amounts of holorganic casts, which are difficult to separate from the ingested substrate [8]. The contact between worm-worked and unworked material may thus affect the decomposition rates, due to the presence of microbial populations in earthworm casts different from those contained in the material prior to ingestion [17], [18]. The inoculum of those communities in fresh organic matter is thus expected to promote modifications similar to those found when earthworms are present, altering microbial community levels of activity and modifying the functional diversity of microbial populations [21], which are key factors for organic matter decomposition.

In addition, the nutrient content of the egested materials differs from that in the ingested material [2], which may enable better exploitation of resources, either because of the appearance of microbial species in the fresh substrate or because of the presence of a pool of readily assimilable compounds in the casts [8]. Indeed, Aira *et al.* [2] found an increase in the labile carbon pool of pig manure in a short-term experiment (72 h) with the epigeic earthworm *E. fetida*; such effects were density-dependent. They detected greater values of dissolved organic carbon (DOC; 2174±253 µg C g−^1^ dw) with the highest density of earthworms (100 earthworms per mesocosm) than that in the control (1146±207 µg C g−^1^ dw); whereas the low and medium earthworm densities (25 and 50 earthworms per mesocosm) showed intermediate values. These authors also observed a strong and linear density-dependent response of N mineralization to the earthworm density, with values of approximately 430 µg N g−^1^ dw in the high earthworm density, reaching up to 1.15 times more than in the control. However, in a mesocosm experiment with the epigeic earthworm *Eudrilus eugeniae*, Aira *et al.* [22] found lower contents of both available C and N forms (DOC and NH_4_^+^) in cast samples (500±30 and 600±20 µg g−^1^ dw, respectively) relative to the initial pig manure, with values of 1,200±60 and 2,020±70 µg g−^1^ dw for DOC and NH_4_^+^ concentrations.

Thakuira *et al.* [23] found that food resource type can cause shifts in the gut wall-associated bacterial community, but the magnitude of these shifts did not obscure the delineation between ecological group specificity. Earthworms of different functional groups, or even different species within the same functional group, have a particular mode of food selection, ingestion, digestion, assimilation and movement, thus their importance in mixing, decomposition or nutrient release, as well as in the structure and activity of microbial communities will vary both qualitatively and quantitatively [24]. However, most of the earlier studies focusing on earthworm-microorganism interactions involve soil-dwelling endogeic (i.e., those earthworms that forage below the surface soil, ingest high amounts of mineral soil and form horizontal burrows) and anecic species (i.e., those that live in deeper zones of mineral soils, ingest moderate amounts of soil, and feed on litter that they drag into their vertical burrows) [23], [25]–[31]; and as such, little is yet known about how the earthworm diet influences the relationships that epigeic earthworms species, which rarely form burrows and feed preferentially on plant litter, establish with microorganisms. In nature epigeic earthworms, such as *Eisenia andrei*, live in fresh organic matter of forest litter, in litter mounds, in herbivore dungs, and in anthropogenic environments such as manure heaps, vegetal debris and vermicomposting beds common in agricultural landscapes [2]. Indeed, the earthworm *Eisenia andrei* is one of the most common epigeic earthworms found in such environments [8], in which the average worm density is around 4950±690 individuals m^−2^ and where the earthworms intensively interact with microorganisms and other fauna within the decomposer community [2], [32], altering the rates of organic matter decomposition. Epigeic earthworms may affect microbial decomposer activity by grazing directly on microorganisms, and by increasing the surface area available for microbial attack after comminution of organic matter [33]. These activities enhance the turnover rate and productivity of microbial communities, thereby increasing the rate of decomposition. These earthworm species may also affect other fauna directly, mainly through the ingestion of microfaunal groups (protozoa and nematodes) that are present within the organic detritus consumed [9]; or indirectly, modifying the availability of resources for these groups [32]. The occurrence of these earthworms at high densities [34] is thus expected to increase their effect on the decomposer community, promoting rapid changes in the structure and activity of microbial communities. In fact, it has been shown that high densities of the epigeic earthworm *E. fetida* promoted an increase in fungal populations after 72 h [2].

The aim of the present study was therefore to investigate whether and to what extent the earthworm *E. andrei* is capable of altering the microbiological properties of fresh organic matter through gut associated processes. We also determined whether these direct effects are related to the earthworm diet, as previously documented for the epigeic/hemiedaphic earthworm *Lumbricus rubellus* [18], which is usually found in moist soils particularly those with a high input of organic matter [34], or if the response is independent of the microbial composition of the parent material. To address these questions we analysed the changes in the microbial community structure (phospholipid fatty acid profiles) and microbial activity (fluorescein diacetate hydrolysis) in the earthworm casts derived from three types of animal manure (cow, horse and pig manure) which differed in microbial composition [35].

## Results

**Figure 1:**
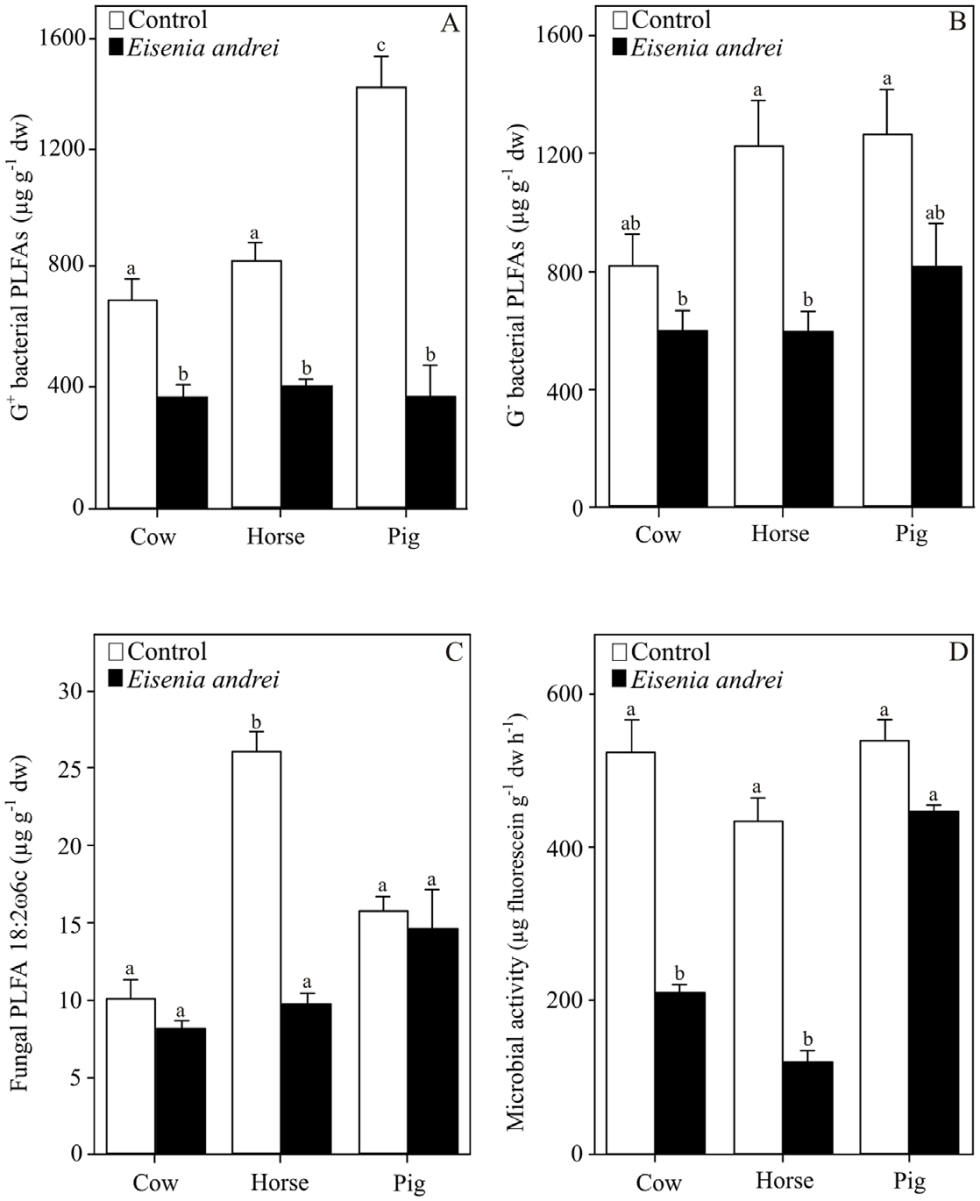
Changes in (A) Gram-positive bacterial PLFAs, (B) Gram-negative bacterial PLFAs, (C) fungal PLFA 18:2ω6c and (D) microbial activity assessed by fluorescein diacetate hydrolysis, after the passage of the three different animal manures (cow, horse and pig) through the gut of the earthworm species *Eisenia andrei*. Values are means ± SE (n = 5). Different letters indicate significant differences between samples (Tukey HSD test; α = 0.05). Controls are the manures incubated without earthworms.

In the present study, twenty-seven phospholipid fatty acids (saturated, mono- and polyunsaturated, and branched PLFAs) ranging from 10 to 20 carbon atoms were identified and quantified in the control and cast samples derived from the three different animal manures (Table 1). Overall, the abundance of Gram-negative bacteria was higher than that for Gram-positive bacteria in all the manures (43 and 38% for G− and G+ bacteria, respectively); although such difference between both microbial groups was more pronounced in cast samples (49 and 28% for G− and G+ bacteria) (Table 1). The passage of the organic material through the gut of the earthworm species *E. andrei* reduced the viable microbial biomass, measured as the total content of PLFAs, relative to the control (Table 1). More specifically, we found up to a 40% reduction in the microbial biomass from all the manure samples (2408±175.7 µg g^−1^ dw) to earthworm casts (1295±98.3 µg g^−1^ dw) (Table 1); although the interaction between the type of manure and earthworm treatment was close to being significant (Table 2). The passage of the organic material through the gut of this earthworm species also led to a reduction in the concentration of both Gram-positive and Gram-negative bacterial PLFAs relative to the control (Figure 1A,B); the effect on G^+^ bacterial PLFAs was more pronounced in pig manure (3.7 times) than in cow and horse manures (1.8 and 2.01 times) (Figure 1A), resulting in a significant interaction between type of manure and earthworm treatment (Table 2). A decrease in fungal biomass, as assessed by the PLFA biomarker 18:2ω6c, was also attributed to the transit of the substrate through the earthworm gut (Figure 1C); this effect depended on the type of manure (Table 2), as the reduction in this PLFA biomarker, relative to the control, was only detected in horse manure (2.7 times; Figure 1C). The transit of the organic material through the gut of *E. andrei* reduced the total microbial activity, measured by fluorescein diacetate (FDA) hydrolysis, in microcosms containing cow and horse manures (2.5 and 3.8 times) (Figure 1D); however, no such reduction was observed in microcosms with pig manure, producing an interaction between type of manure and earthworm treatment (Table 2).

**Table 1:** PLFA yields (µg g^−1^ dw) in control and cast samples derived from the three types of animal manure (cow, horse and pig).

	Cow manure		Horse manure		Pig manure	
	Control	*E. andrei*	Control	*E. andrei*	Control	*E. andrei*
PLFA biomarkers						
*G+ bacteria*						
i14:0	59.3±6.9	22.3±3.1	76.9±5.1	25±0.9	117.9±9.2	15±3.8
i15:0	296±24.4	195±18.2	406.6±23	192±8.5	485.9±27.2	174.3±33
a15:0	171±12.6	83.8±8.5	157.3±6.9	90.7±4.5	406.7±19.6	83.5±17.3
i16:0	67.8±7.3	41.04±5.2	88.4±5.0	46.8±1.9	165.5±12.9	42.8±8.8
a17:0	74.9±6.7	46.0±5.9	95.6±10.2	49.6±2.3	173.5±43.4	53.8±11.1
*G− bacteria*						
16:1ω7c	215.7±18	150.3±22.3	226±62.3	125.7±11.0	191.9±21.6	161.1±25.3
17:1ω7c	92.0±7.8	50.6±6.5	144±19.6	60±3.6	125.3±23.6	57.1±16.2
18:1ω7c	462.8±118	358.3±125.5	814±107.9	388.5±82.2	899.7±39.9	561.8±115.1
cy17:0	20.2±2.1	18.3±4.8	23.3±3.4	19.1±6.7	21.5±2.3	18.6±2.6
cy19:0	15.4±3.1	15.0±1.8	16.6±0.6	15.3±2.4	15.3±3.1	15.1±1.5
*Fungi*						
18:2ω6c	10.1±1.5	8.0±1.5	26.2±0.8	9.8±1.6	15.8±0.8	15.4±2.9
Other microbial PLFAs						
10:0	0.8±0.03	0.9±0.1	1.0±0.07	1.3±0.04	1.2±0.03	0.8±0.03
12:0	4.9±0.4	5.7±0.6	7.1±0.4	7.7±0.6	6.1±0.5	4.5±0.9
13:0	1.7±0.2	2.4±0.4	1.6±0.3	3.3±0.3	2.6±0.5	2.2±0.6
14:0	31.6±2.9	19.0±3.1	42.4±3.5	21.3±1.7	54.5±0.5	21.2±4.5
15:0	25.7±2.5	15.0±1.3	27.9±2.1	15.6±1.6	29.7±1.1	15.9±3.3
16:0	166.4±11.0	115.0±20.2	199±19.8	117.1±8.7	210.3±22.8	144.0±26.9
17:0	37.5±4.3	37.5±3.5	35.1±4.8	36.3±5.6	43.7±2.8	38.4±8.1
18:0	12.6±0.3	15.8±0.6	12.7±1.0	15.3±0.6	15.9±0.9	16.7±2.9
14:1ω5c	1.3±0.1	0.4±0.01	0.5±0.02	0.6±0.1	5.8±0.3	1.1±0.3
15:1ω5c	0.5±0.01	0.9±0.02	0.8±0.2	4.3±0.7	7.6±1.7	1.4±0.6
18:1ω9c	13.2±1.8	8.4±2.2	15.0±2.0	10.9±0.5	23.2±0.6	13.5±2.6
18:1ω9t	1.2±0.1	1.0±0.2	1.3±0.4	1.0±0.0	1.5±0.3	1.4±0.2
18:2ω6t	1.0±0.1	1.6±0.1	1.2±0.1	2.5±0.9	1.1±0.1	3.4±0.7
18:3ω3c	3.1±0.6	1.8±0.4	4.2±0.1	1.7±0.2	1.4±0.1	1.2±0.1
18:3ω6c	0.8±0.1	0.7±0.1	1.7±0.01	0.8±0.02	0.9±0.01	0.9±0.1
20:0	2.1±0.1	2.0±0.1	2.4±0.2	2.3±0.1	3.4±0.3	4.3±0.9
Total PLFAs	1790±220.2	1217±189.6	2405±280	1199±126.5	3028±164.9	1469±267

Values are means ± standard error (n = 5).

**Table 2:** ANOVA for total, Gram-positive, Gram-negative and fungal PLFAs and fluorescein diacetate (FDA) hydrolysis for the three types of animal manure (cow, horse and pig) incubated with and without earthworms.

	Manure type		Earthworm treatment		Manure×Earthworm	
	*F*_2,24_	*p*	*F*_1,24_	*p*	*F*_2,24_	*p*
**Total PLFAs**	7.81	0.002	50.71	0.000	3.32	0.054
**G^+^ bacterial PLFAs**	15.10	0.001	116.89	0.000	16.74	0.000
**G^−^ bacterial PLFAs**	3.94	0.033	18.39	0.000	1.39	0.267
**Fungal PLFAs**	17.88	0.000	27.60	0.000	15.41	0.000
**FDA hydrolysis**	18.84	0.000	62.13	0.000	5.07	0.015

Five replicates were included per treatment.

**Figure 2:**
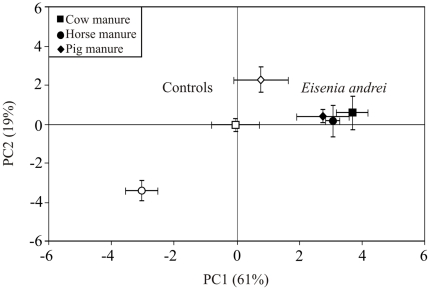
Changes in microbial community structure after the passage of the three different animal manures (cow, horse and pig) through the gut of the earthworm species *Eisenia andrei*. Principal component analysis performed on the twenty-seven PLFAs identified in cast (filled symbols) and control (open symbols) samples derived from the different animal manures. The first and second principal components (PC1 and PC2) explain 61 and 19% of the variance respectively. Values are means ± SE. Controls are the manures incubated without earthworms.

The passage of the organic material through the gut of the earthworm *E. andrei* strongly modified the structure of microbial communities in all three types of animal manure, as revealed by the principal component analysis (Figure 2). Indeed, a clear separation between cast and control samples was found along the first principal component (PC1; accounting for 61% variance); this effect was more pronounced in horse manure than in cow and pig manures (Figure 2), resulting in a significant interaction between type of manure and earthworm treatment (*F*_2,24_ = 4.003, *p* = 0.03). The second function also contributed in differentiating cast from control samples in microcosms with horse and pig manures (*F*_2,24_ = 26.47, *p* = 0.000), accounting for 19% of the observed variance (Figure 2). Moreover, control samples derived from the different animal manures were clearly differentiated from each other (Figure 2); while no such distinction was found for cast samples in both principal components (Figure 2).

## Discussion

In this study, we found that the transit of fresh organic matter through the gut of *E. andrei* strongly modified the structure and activity of microbial communities, revealing the existence of important direct effects of this earthworm species on the microbial decomposers, i.e. bacteria and fungi. Recent reports suggest that the digestion of organic material by epigeic earthworms has negative effects on microbial biomass [10], [14], [22]. The present data are consistent with these findings, since we found a reduction in the viable microbial biomass as a result of the passage of the fresh substrate through the earthworm gut. Epigeic earthworms possess a diverse pool of digestive enzymes which enables them to digest bacteria, protozoa, fungi and partly decomposed plant debris [36]. Indeed, bacterial populations decreased in the three types of animal manure after transit through the earthworm gut. More specifically, earthworm activity reduced the abundance of G+ bacteria to a greater extent than G− bacteria (2.5 and 1.6 times respectively). Such differences may be due to the fact that G− bacteria possess an outer membrane composed of lipopolysaccharides, which provides them with structural integrity increasing the negative charge of the cellular membrane and protecting them against certain types of chemical attack [37]. Previous studies involving the effects of epigeic earthworms on microorganisms have also shown that G− bacteria can survive the transit through the earthworm gut [38]–[40].

In contrast to the decrease in bacteria, no such reduction was found for fungal populations in microcosms containing cow and pig manure. The impact on fungal populations was more pronounced in horse manure, probably because the values were already higher in this type of manure before the addition of earthworms. Animal manures are microbial-rich environments in which bacteria constitute the largest fraction (around 70% of the total microbial biomass as assessed by PLFA analysis), with fungi mainly present as spores [8]. Thus, earthworm activity is expected to have a greater effect on bacteria than on fungi in these organic substrates. Domínguez *et al.* [8] reported a reduction in the bacterial growth rate of cow manure after having been processed by *E. andrei* for one month, whereas no such decrease was detected for fungal growth, which supports the idea that in this type of organic substrate, the latter microbial group is less affected by the passage through the earthworm gut. Similarly, Aira *et al.* [2] detected a significant increase in the fungal biomass of pig manure, measured as ergosterol content, in a short-term experiment (72 h) with the epigeic earthworm *E. fetida*, and the effect depended on the density of earthworms. A higher fungal biomass was found at intermediate and high densities of earthworms (50 and 100 earthworms per mesocosm, respectively), which suggests that there may be a threshold density of earthworms at which fungal growth is triggered. This priming effect on fungal populations was also observed in previous short-term experiments in the presence of the epigeic earthworms *Eudrilus eugeniae* and *Lumbricus rubellus* fed with pig and horse manure, respectively [22], [35]. Collectively, the aforementioned studies indicate that epigeic earthworms favour the fungal growth in this type of organic substrates in the short-term. These contrasting short-term effects on bacterial and fungal populations are thus expected to have important implications on decomposition pathways because there exist important differences between both microbial decomposers related to resources requirements and exploitation [41]; and as such, if the earthworms were to depress bacteria whilst leaving the fungi unaffected in the egested materials, earthworms are expected to greatly affect nutrient cycling and decomposition rates. Indeed, both microbial decomposers support their own decomposition pathway in soil, and in turn fungal and bacterial biomass production will cascade through different channels of the terrestrial ecosystem. Moore & Hunt [42] proposed that bacterial and fungal energy channels have distinct functions, representing fast and slow cycles of nutrient availability, respectively. Therefore, ecosystems dominated by bacterial channels are characterized by a high nutrient availability and low amounts of nutrient-rich organic matter; whilst those dominated by fungal channels are mainly characterized by a high organic matter content and low resource availability and quality [41]. This is based on the fact that fungi can immobilize great quantities of nutrients in their hyphal networks, whereas bacteria are more competitive in the use of readily decomposable compounds and have a more exploitative nutrient use strategy by rapidly using newly produced labile substrates [43]. In addition, bacterial tissues have a lower carbon-to nutrient ratio and their biodegradability is higher relative to the fungal tissues, and in turn bacterial tissues are consumed by their predators to a much greater extent, resulting in a greater nutrient release and turnover [41].

Similarly, Aira *et al.* [44] reported an increase in fungal biomass in a long-term experiment (36 weeks) in the presence of *E. fetida* fed with pig manure. They found that the fungal biomass, measured as ergosterol content peaked after four weeks of vermicomposting, reaching up 7.5 times more than in the control; this priming effect on fungal populations with earthworm presence appeared to be permanent during the process, although the ergosterol content decreased over time, with values ranging between 20 and 50 µg g^−1^ dw. In addition, Aira *et al.* [44] found that such increase in fungal biomass was accompanied by a higher rate of cellulose decomposition in the presence of earthworms. Indeed, these authors observed that the rate of cellulolysis was two times higher relative to the control, resulting in a 1.5-fold increase of cellulose loss after 18 weeks.

Although viable microbial biomass was lower after earthworm gut transit, irrespective of the type of manure, no such decrease was detected in the microbial activity of cast samples derived from the pig manure. Similarly, Aira & Domínguez [45] did not find any changes in microbial activity in casts of *Eisenia fetida* fed with pig manure. Aira *et al.* [10] also did not detect any significant differences between the initial pig manure and the gut contents of three epigeic earthworm species (*Eisenia andrei*, *Eisenia fetida* and *Eudrilus eugeniae*) in terms of dehydrogenase activity. The latter authors used the same food resource (i.e., pig manure) for all the three earthworm species in order to avoid masking effects in their response to ingested microflora due to differences in the nutrient quality and/or substrate availability. As such, unlike in the study of Aira *et al.* [10], the present work demonstrated that the composition of the parent material played a key role in the relationships between earthworms and microorganisms in terms of microbial activity, because similar values of basal respiration were found in cast samples derived from cow and horse manures; whereas, in microcosms with pig manure, microbial activity was found to be about two and four times higher, relative to the former manures, after transit through the earthworm gut. Collectively, the aforementioned studies indicate that the remaining microbes are still able to maintain a high level of metabolic activity, which may lead to further degradation of the pig manure. Indeed, previous studies of the effects of epigeic earthworms on microorganisms have shown that the passage of microorganisms through the earthworm gut may result in a smaller but metabolically more active microbial population. More specifically, Gómez-Brandón *et al.* [46] found that the activity of the epigeic earthworm *Eisenia fetida* reduced the bacterial and fungal biomass by approximately 1.4 and 1.2 times relative to the control during decomposition of pig manure; such effect on bacterial populations was dependent on the rate of detritus input. Despite having reduced the microbial biomass, the pronounced presence of earthworms stimulated the levels of activity of microbial communities during the first stages of decomposition (i.e., after 2 weeks of vermicomposting), reaching up 1.5 and 1.1 times more than in the control, for low and high inputs of organic matter (1.5 and 3 kg of pig manure, respectively). The above-mentioned changes resulted in alterations of the system functioning, as shown by the greater loss of total carbon with earthworm presence (440 and 200 µg C day^−1^, with and without earthworms). As mentioned before, by enhancing fungal growth, the earthworm E. *fetida* also triggered more efficient cellulose degradation [44], which reinforces the fact that epigeic earthworms play a critical role in organic matter decomposition because of the interactions they establish with microorganisms. In addition to a quick carbon mineralization, epigeic earthworms have been shown to promote a change in the functional diversity of microbial communities increasing their capabilities to use more diverse carbon pools [3]. This suggests that microbial communities use the available energy more efficiently in the presence of earthworms; and in turn the system functions much better, as shown by the large increase in the rate of organic matter decomposition [3], [44], [46]. Recently, Aira & Domínguez [21] found that the inoculation of raw organic matter (i.e., pig manure) with worm-worked material (i.e., vermicompost) also modified microbial community functioning, altering microbial community levels of activity. As discussed above, microbial communities from worm-worked material (i.e., casts and/or vermicompost) are metabolically more diverse and also have higher levels of microbial activity than those in the unworked material [3], [21], [44], [46], and in turn the presence of and interaction between microbial communities from these different processed materials is expected to enhance decomposition rates. Such indirect effects of earthworms on organic matter decomposition are independent of the amount of worm-worked substrate inoculated, suggesting the existence of a threshold at which functioning is triggered [21]. The strength of the process greatly depends on the type of vermicompost used [21], indicating that the earthworm species is a major factor affecting earthworm-microorganism interactions. Such earthworm-specific effects are consistent with the fact that specific microbial groups respond differently to the gut environment, depending on the earthworm species [47].

Vermicomposting systems sustain a complex food web [7], in which detritivore earthworms interact intensively with microorganisms and other fauna within the decomposer community, accelerating the stabilization of organic matter and greatly modifying its physical and biochemical properties [8]. Stabilisation involves the decomposition of an organic material to the extent of eliminating the hazards and is normally reflected by decreases in microbial biomass and its activity and concentrations of labile compounds [48]. Overall, in the present study a higher degree of stabilisation was reached in the organic substrates once they were released as casts, since lower values of microbial biomass and activity which are indicative of stabilised materials, were found after passage of the substrates through the earthworm gut. Similarly, previous studies have already shown the potential of epigeic earthworms in the stabilisation of this type of organic substrates in the short-term [22], [45]. For instance, Aira *et al.* [22] observed that the values of microbial biomass carbon and microbial activity, measured as basal respiration were much lower in casts of *E. eugeniae* (5,100±200 µg g^−1^ dw and 350 µg CO_2_ g^−1^ OM h^−1^, respectively) relative to the initial pig manure (8,900±700 µg g^−1^ dw and 980 µg CO_2_ g^−1^ OM h^−1^). In addition, Aira & Domínguez [45] also found lower values of microbial activity, measured as basal respiration in casts of *E. fetida* (510 µg CO_2_ g^−1^ OM h^−1^) than in the initial cow manure (920 µg CO_2_ g^−1^ OM h^−1^). This is of great importance to the application of animal manures as organic amendments into agricultural soils. It is widely recognised that the overproduction of this type of organic substrates has led to inappropriate disposal practices, which may result in severe risks to the environment such as an excessive input of potentially harmful trace metals, inorganic salts and pathogens; increased nutrient loss from soils and the emission of toxic gases [48]. Thus, with the present study, we showed that earthworms may favour the stabilisation of the organic matter of these types of substrates via their effects on microbial communities through the gut associated processes; thereby contributing to minimize the potential risks related to the land-spreading of manures into soil.

Several previous studies have shown changes in the structure of microbial communities as a consequence of the passage of organic material through the earthworm gut [18], [49]–[50]. However, the present results are unique in that although the three types of animal manure differ in their microbial composition, there were no differences between cast samples derived from the different types of manure with regards to bacterial and fungal biomass and microbial community structure. This provides strong evidence that the transit of organic material through the gut of *E. andrei* exerts a bottleneck effect on microbial communities, suggesting that the direct effects of epigeic earthworms on the microbial community structure are largely determined by factors other than the parent material. This is consistent with the results of a previous study in which we observed that the earthworm species *E. andrei* created similar structures of microbial communities in vermicomposts produced after one month from different starting materials [35]. Therefore, the previous and the present study together indicate that the earthworm gut is a major shaper of microbial communities, acting as a selective filter for the microorganisms contained in the ingested material. Indeed, these results point to biotic interactions between epigeic earthworms and microorganisms through the gut associated processes as important drivers of organic matter decomposition and nutrient cycling, by altering the levels of activity of microbial communities; and thereby favouring the existence of a reduced but more active microbial community specialized in metabolizing compounds produced or released by the earthworms, in the egested materials. This suggests that the passage of organic material through the earthworm gut may modify the functional diversity of microbial populations and promote a more efficient use of the available energy by microorganisms of the ingested material; which is expected to have important implications in regulating nutrient cycling in terrestrial ecosystems, thus probably enhancing decomposition rates and nutrient turnover.

Overall, the present study provides insight into the direct effects of epigeic earthworms on the microbial decomposers, and further illustrates the important role of these earthworm species in shaping the structure of microbial communities through gut associated processes. Therefore, it will be of future interest to determine whether the changes in the composition of the microbiota in response to the earthworm diet are accompanied by a change in the microbial diversity and/or functioning in the egested materials. Ultimately, this knowledge will help us to better understand the functional importance of earthworms on soil decomposition processes and overall soil function, which is of great importance to understand how biotic interactions within the decomposer food web influence on nutrient cycling.

## Materials and Methods

### Ethics statement

No permits were required for the collection of animal manures as the farm in question collaborates with the University of Vigo providing manure without requiring remuneration for doing so.

### Experimental material and set up

The animal manures were collected from a farm near the University of Vigo (Galicia, NW Spain). Specimens of *E. andrei* were collected from a stock culture reared under laboratory conditions (20±2°C), and were starved for two days prior to the experiment in order to ensure that their guts were empty.

The microcosms consisted of plastic containers (250 mL) filled to three quarters of their capacity with sieved, moistened vermiculite and inoculated with 25 mature earthworms. Vermiculite is a hydrated silicate mineral resembling mica and does not contain any organic nutrients, which thus obliged the earthworms to ingest the substrate provided. A plastic mesh was placed over the surface of the vermiculite and 100 g (fresh weight, fw) of the substrate was placed on top of the mesh, to avoid the substrate becoming mixed with the vermiculite bedding. The microcosms were covered with perforated lids and stored in random positions in an incubation chamber, at 20°C and 90% relative humidity, for three days. Control microcosms consisted of each type of manure incubated without earthworms. Each treatment was replicated five times. In order to obtain cast samples, earthworms were removed from the microcosms, washed three times with distilled water and placed in Petri dishes on moistened filter paper. Casts from the same Petri dish were then collected with a sterile spatula and pooled for analysis in 1.5 mL Eppendorf tubes; the same amount of manure samples were also collected from the control microcosms. All samples were immediately stored at −20°C for phospholipid fatty acid (PLFA) analysis and at 4°C for determining microbial activity, assessed by hydrolysis of fluorescein diacetate (FDA).

### Determination of microbial community structure by PLFA analysis

Analysis of phospholipid fatty acid (PLFA) composition is one of the most commonly used culture-independent tools for investigating microbial populations in ecological studies [51]. This methodology provides qualitative and quantitative insights into the structure of the microbial community and indicates the main groups of microorganisms present and their abundance. The biggest strength of this lipid-based approach, as compared to other microbial community assays, is that PLFAs are rapidly synthesised during microbial growth and quickly degraded upon microbial death and they are not found in storage molecules, thereby providing an accurate ‘fingerprint’ of the current living community [52].

The total lipidic extract was obtained from 200 mg of each freeze-dried sample with 60 mL of chloroform-methanol (2∶1, v/v), as described by Gómez-Brandón *et al.* [53]. Lipid fractionation was performed by solid phase extraction (SPE). This technique concentrates and purifies the analytes from a solution by adsorption on a solid phase, followed by elution of the analytes with an appropriate solvent for further analysis. Silica-based sorbents are the most commonly used in SPE; silanols, active sites located in the granules of silica acid, have hydroxyl groups attached directly to the silicon atom, and interact with the polar groups of the various lipid classes, while the non polar end of the lipid molecule contributes to their separation. Using solvents of increasing polarity (chloroform<acetone<methanol) the selective elution of the different lipid classes is achieved. In the present study, the lipid extract was then fractionated into neutral lipids, glycolipids and phospholipids with chloroform (5 mL), acetone (10 mL) and methanol (5 mL), respectively, on silicic acid columns (Strata SI-1 Silica (55 µm, 70 Å), 500 mg/6 mL). The methanolic fraction containing phospholipids was evaporated under an O_2_-free N_2_ stream and subjected to derivatization with trimethylsulfonium hydroxide (TMSH) at room temperature to obtain the fatty acid methyl esters (FAMES), following the protocol of Batista *et al.* [54]. Briefly, phospholipid extracts were redissolved in 500 µL of methyl-*tert*-butyl ether. One hundred microliters of this solution were placed in a screw-cap vial with 50 µL of the derivatizating agent (TMSH), vortex-mixed for 30 s and allowed to react for 30 min; 10 µL of the internal standard methyl nonadecanoate (19∶0, 230 µg mL^−1^) was then added to the extract of FAMEs prior to gas chromatography-mass spectrometry (GC-MS) analysis. The detailed GC-MS experimental conditions are described elsewhere [53]. To identify the FAMEs, the retention times and the mass spectra were compared with those obtained from known standard mixtures or pure PLFAs. FAMEs were quantified by an internal standard calibration procedure [53]. The calibration levels of the FAMEs varied in the range 0.4–250 µg mL^−1^. The coefficients of determination (R^2^) were higher than 0.99 for all calibration curves. FAMEs were described by the standard ω-nomenclature A:BωC [55].

The sum of all identified PLFAs (total PLFAs) was used to estimate the viable microbial biomass [56]. Certain PLFAs were used as biomarkers to determine the presence and abundance of specific microbial groups [56]. The sum of PLFAs characteristic of Gram-positive (i14:0, i15:0, a15:0, i16:0 and a17:0), and Gram-negative bacteria (16:1ω7c, 17:1ω7c, 18:1ω7c, cy17:0 and cy19:0) were chosen to represent bacterial PLFAs, and the PLFA 18:2ω6c was used as a fungal biomarker.

### Determination of microbial activity by FDA hydrolysis

Fluorescein diacetate (FDA) is a colourless fluorescein conjugated that is hydrolysed by both free (exoenzymes) and membrane bound enzymes [57], releasing a coloured end product, fluorescein. The enzymes responsible for FDA hydrolysis, such as non-specific esterases, proteases and lipases are involved in the decomposition of organic matter in soil. The ability to hydrolyse FDA thus seems widespread, especially among the major decomposers, bacteria and fungi [57]. Since more than 90% of the energy flow in a soil system passes through microbial decomposers, and heterotrophic microorganisms are predominant in soil, FDA hydrolysis is thought to reflect the overall soil microbial activity [58].

Fresh samples (0.5 g, fresh weight) were incubated for 20 min at 30°C after the addition of 3.75 mL of 60 mM phosphate buffer (pH 7.6) and 0.05 mL of a 1000 µg mL^−1^ solution of FDA prepared in acetone. The reaction was stopped by adding 3.75 mL of chloroform and methanol (2∶1, v∶v). Samples were then centrifuged at 2000 rev min^−1^ for 3 min and the aqueous phase filtered (Whatman, No. 2). Absorbance of the filtrates was measured within the next 50 min at 490 nm in a Bio-Rad Microplate Reader 550 [58].

### Statistical analysis

Data were analyzed by ANOVA, with the type of manure (cow, horse and pig) and earthworm treatment (presence and absence) as the main factors. Significant differences in the main effects were further analyzed by paired comparisons with the Tukey HSD test. A principal component analysis was also used to analyze the PLFA data in order to assess overall differences in the microbial community structure of the three types of animal manure after passage through the gut of the earthworms. The PCA scores were analyzed by ANOVA and the Tukey HSD test, as above. All statistical analyses were performed with Statistica software (version 7).
